# Characteristics and geographical distribution of syphilis among people with human immunodeficiency virus and the National Population in Republic of Korea

**DOI:** 10.1371/journal.pone.0340324

**Published:** 2026-03-26

**Authors:** Yae Jee Baek, Seyoung Kim, Eunjung Lee, Boyoung Park, Oeuk Jeong, Jongtak Jung, Jun Yong Choi, Tae Hyong Kim

**Affiliations:** 1 Division of Infectious Diseases, Department of Internal Medicine, Soonchunhyang University Seoul Hospital, Soonchunhyang University College of Medicine, Seoul, Republic of Korea; 2 Institute for Health and Society, Hanyang University, Seoul, Republic of Korea; 3 Department of Public Health Science, Graduate School of Public Health, Seoul National University, Seoul, Republic of Korea; 4 Department of Preventive Medicine, College of Medicine, Hanyang University, Seoul, Republic of Korea; 5 Division of Clinical Research, National Institute of Health (NIH), Osong, Republic of Korea; 6 Department of Internal Medicine, Severance Hospital, Yonsei University College of Medicine, Seoul, Republic of Korea; University of Westminster - Regent Street Campus: University of Westminster, UNITED KINGDOM OF GREAT BRITAIN AND NORTHERN IRELAND

## Abstract

**Introduction:**

The global incidence of syphilis has been increasing in recent years, with multiple factors contributing to this trend. Syphilis disproportionately affects specific populations. We aimed to evaluate trends in the incidence and geographical distribution of syphilis at the provincial level among people living with human immunodeficiency virus (PLWH) and the total population in Korea.

**Methods:**

PLWH were enrolled from the Korea National Health Insurance Service Database. Early syphilis was defined as having an International Classification of Diseases, 10^th^ revision diagnostic code and the prescription codes for medications. Between 2014 and 2019, syphilis was designated as a nationally notifiable infectious disease, facilitating comprehensive data collection. We compared the number of syphilis cases in the total population and the PLWH cohort. The incidence rates of early syphilis were estimated using Poisson regression, complemented by provincial mapping among the national and PLWH populations.

**Results:**

The prevalence of early syphilis among PLWH was 22.4%, with 58.6% occurring after HIV diagnosis. In the total population, primary syphilis accounted for 69.8% of early cases. The age distribution and regional epidemiology of syphilis among PLWH differed from those in the total population. HIV infection was associated with a 5.04-fold higher incidence rate of syphilis (Incidence rate ratio (IRR), 95% Confidence interval (CI): 5.04 [4.77–5.34]), and as the years elapsed, the incidence rate of syphilis increased by 14.5% (IRR, 95% CI: 1.15 [1.13–1.16]). Since 2015, the incidence of new syphilis cases has increased notably in the total population, whereas it remained relatively stable among PLWH, indicating that the absolute rate and trajectories differed from those of the national population.

**Conclusion:**

Syphilis is prevalent among PLWH; however, the epidemiology differs from that of the national population. This highlights the need for multifaceted policies to prevent syphilis transmission.

## Introduction

Syphilis is a sexually transmitted infection (STI) caused by the spirochete *Treponema pallidum* subspecies *pallidum*. If left untreated, hematogenous dissemination may occur, leading to multi-organ involvement and late-stage complications [1]. Historically, syphilis was a major public health threat due to its high transmissibility and severe clinical sequelae—including congenital syphilis—until the introduction of penicillin in the 1940s, after which the incidence declined substantially. However, many countries have documented a resurgence since the early 2000s [2,3]. The World Health Organization estimates that approximately eight million adults aged 15–49 years acquired syphilis in 2022, and congenital syphilis remains a leading cause of stillbirth in low- and middle-income countries. Furthermore, several countries have reported increases in adult and congenital syphilis after the COVID-19 pandemic [4,5],

Multiple factors are thought to contribute to this resurgence. Population-level increases have been attributed to shifts in sexual networks and contact patterns, including the use of digital platforms to seek partners [6]. Limited-resource settings face persistently high congenital syphilis rates due to barriers in diagnostic access [7–9]. In several high-income countries, disproportionate increases among men who have sex with men (MSM) have been described [10].

Syphilis and human immunodeficiency virus (HIV) share transmission routes, and epidemiological studies have demonstrated an association between incident syphilis and HIV infection [11]. People living with HIV (PLWH) may experience a higher burden of syphilis [12]. This may be attributed to overlapping risk behaviors, network effects, and potential biological susceptibility, although the magnitude and mechanisms vary across settings [13]. There are concerns that using HIV pre-exposure prophylaxis, a key strategy in HIV management, may lead to compensation for risky sexual behaviors and an increase in STIs [14,15]. In the United States, incident syphilis has been associated with women of childbearing age who use illicit drugs and PLWH [11]. In Türkiye, although HIV/syphilis co-infection appeared relatively high among heterosexuals, the number of cases among MSM showed a moderate increase [16]. Nevertheless, few studies have directly quantified the correlation between syphilis trends and PLWH on a national scale.

Robust monitoring through national surveillance systems is essential to describe trends, epidemiology, and risk factors, and inform targeted interventions (such as STI prevention education, antenatal screening, and resource allocation). Given the complexity of transmission dynamics and limitations inherent to any single data source, triangulating multiple datasets can improve inference. Therefore, in this study, we aimed to evaluate trends in the incidence and geographical distribution of syphilis at the provincial level among the total population and PLWH.

## Methods

### Landscape of syphilis surveillance system and Sources of data

In this study, we used the Korean National Health Insurance Service (NHIS) and the Korean Disease Control and Prevention Agency (KDCA) Infectious Disease Surveillance datasets. The NHIS database, available since 2002, is widely used for population-level studies because of its wide range and detailed health, demographics, and medical services records covering nearly the entire population. By the end of 2020, health security benefits had been extended to 52,870,000 individuals in South Korea. The NHIS covered 51,340,000 of these individuals, representing 97.1% coverage, while the remaining 1,530,000 were medical aid beneficiaries. Diagnoses are coded according to the Korean Standard Classification of Diseases, which is based on the International Classification of Diseases (ICD) [17]. NHIS claims data from 2002 to 2019 were used in this study.

Syphilis surveillance in Korea began as a sentinel (sample-based) system in 2001 and was expanded into a comprehensive, nationwide notifiable system from 2010 to 2019, in line with statutory classification updates. In 2020, following a reorganization of infectious-disease classes, syphilis was designated Class 4 and returned to sentinel surveillance [18]. In January 2024, syphilis was upgraded to Class 3, reinstating nationwide notification in response to rising incidence in neighboring countries and the imperative to eliminate congenital syphilis [19]. Under the statutory national-notifiable system, designated reporters (including physicians and laboratories) are legally required to notify local health authorities immediately or within 24 h. This prompt reporting facilitates thorough epidemiological investigations and effective monitoring of disease outbreaks [19]. For this study, we used KDCA national notifiable surveillance data for 2011–2019. Using the KDCA annual infectious-disease surveillance yearbooks, we obtained annual counts of newly reported syphilis cases. Detailed epidemiologic investigation forms, available from 2014 onward, were obtained through an official request from the Division of HIV/AIDS Prevention and Control, KDCA (accessed February 17, 2024).

### Definitions and study period

In the NHIS dataset, the initial study population comprised 20,423 individuals who received the catastrophic illness registration code V103 for HIV infection at least once. HIV was defined as having received the V103 code at least twice. We excluded individuals diagnosed between 2002 and 2004 to minimize potential overestimation during the initial observation years (washout period). Those whose first HIV diagnosis was made after 2020 were excluded. The date of the first HIV diagnosis was based on the first claim date associated with V103 or ICD-10 codes (B20, B21, B22, B23, and B24). New syphilis cases were identified using the ICD-10 codes (A50, A51, A53) with a prescription of a single dose of Benzathine Penicillin G, a 14-day prescription of doxycycline, or ceftriaxone 1g for 10–14-days.

The study period spanned from 2014 to 2019, during which the two datasets could be compared and merged. During this period, statutory notifications for syphilis were required only for cases classified as primary or secondary syphilis, based on a comprehensive assessment of clinical findings and serologic test results. The analysis was aligned with the period of full national statutory surveillance to ensure comparability with the PLWH cohort, whereas data from 2020–2023 corresponded to sentinel surveillance during the COVID-19 pandemic and were excluded. Therefore, for 2011–2013, we analyzed only overall case counts and proportions, whereas age- and province-specific analyses were restricted to 2014–2019.

### Variables

The PLWH cohort defined in this study was dichotomized into those diagnosed with early syphilis and those who were not. Age at first HIV diagnosis was categorized into the following intervals: < 20, 20–29, 30–39, 40–49, 50–59, and ≥60 years. The age at HIV diagnosis and sex were included in this analysis. Income levels were classified into five groups: medical aid beneficiaries and quartiles (Q1, Q2, Q3, and Q4). The KDCA dataset provided basic patient details, such as sex, age, diagnosis and report dates. Syphilis was categorized as primary, secondary, and congenital. Both datasets included residential areas coded as eight cities and nine provinces.

### Ethics statement

The Institutional Review Board (IRB) of Severance Hospital in the Yonsei University Health System approved this study (Approval No 4-2022-0676). The research was registered on the website to obtain permission to use and analyze the dataset. The informed consent requirement was waived because of the nature of the public data provided by the NHIS. All analyses were performed using de-identified NHIS and KDCA surveillance data with no direct identifiers accessible to investigators, and confidentiality and anonymity were ensured under IRB approval.

### Statistical analysis

We conducted a frequency analysis to understand the population characteristics and compared the differences in categorical variables using the chi-square test. Statistical significance was set at p < 0.05 on both sides. The incidence of syphilis was calculated by comparing the syphilis incidence rate among PLWH identified in the NHIS dataset and the total population in the KDCA nationwide surveillance data. The crude incidence rate was calculated by dividing the number of syphilis cases diagnosed among PLWH by the total number of PLWH during the study period. Meanwhile, for the national population, the crude incidence rate was calculated by dividing the number of syphilis cases identified through nationwide surveillance by the mean population over the same period. Demographic information for the total population was obtained from the national census, conducted annually by public institutions and managed by the National Statistical Office. Age- and sex-standardized incidence rates (per 100,000 persons) were calculated using the direct standardization method. Data were stratified by year, sex, age group, and syphilis type (primary or secondary). The 2020 Korean resident registration population from Statistics Korea was used as the standard population; the proportion of each age–sex group in this population was applied as a weighting factor. Standardized rates were derived by summing the weighted age-specific rates for each year and sex, allowing comparison of incidence across years while accounting for demographic differences. Both incidence rates were expressed as cases per 1,000 population, and the calculated rates were visualized on geographical maps, with darker colors indicating higher incidence rates.

Choropleth maps were generated at the provincial level using population census data and calculated syphilis incidence rates. In addition, Shapefiles representing administrative boundaries were sourced from the Address Information Industry Support Service website of the Ministry of the Interior and Safety and used to map the incidence rates per 1,000 population for each region. The base map was created using publicly available shapefile data provided by the Ministry of Land, Infrastructure and Transport, Republic of Korea. These data are freely available for public use and contain no copyright restrictions. Color gradients were applied to represent varying incidence levels, highlighting geographical differences in syphilis rates using RStudio.

Factors influencing the incidence of syphilis including HIV status, year of diagnosis, and sex were analyzed using Poisson regression analysis. All statistical analyses were performed using SAS statistical software (version 9.4; SAS Institute, Cary, NC, USA), and all figures were generated using RStudio (Posit Software, Boston, MA, USA).

## Results

### Epidemiology of syphilis among PLWH enrolled in the NHIS dataset

In the HIV dataset, 20,423 PLWH were enrolled in the Korea NHIS Database between 2002 and 2021. Of the eligible individuals, 3,423 were excluded due to incomplete diagnoses and washout periods. We excluded 1,861 PLWH because their first diagnosis was not between 2004 and 2019. The study population comprised 14,833 individuals.

Of these, 3,317 (22.4%) were confirmed to have syphilis; 1,373 (41.4%) were diagnosed before HIV infection, and 1,944 (58.6%) were diagnosed afterward (S1 Fig). Table 1 shows the demographic and clinical characteristics of PLWH stratified by syphilis. The PLWH in the incident syphilis group had a higher proportion of males, and a greater proportion of individuals diagnosed with HIV in their 20s and 30s. In addition, the economic status was similar between individuals with syphilis and those without syphilis. Compared to PLWH who had not been diagnosed with syphilis, those with syphilis were more likely to have received an earlier HIV diagnosis, although it was not statistically significant. Regionally, a higher proportion of cases was observed in Seoul (37.5%) and Gyeonggi-do (20.8%). PLWH with early syphilis were more likely to reside in Seoul (37.5% vs. 31.3%) and Incheon (7.2% vs. 5.9%) than those without early syphilis.

**Table 1:** Demographics and clinical characteristics of people living with HIV (PLWH) stratified early syphilis infection.

	PLWH without early syphilis (n = 11,516)	PLWH with early syphilis (n = 3,317)	p-value
Sex			<0.001
Male	10,230 (88.83)	3,286 (99.07)	
Female	1,286 (11.17)	31 (0.93)	
Age of first diagnosis of HIV			<0.001
Below 20-year-old	312 (2.71)	81 (2.44)	
20–29 year-old	2,855 (24.79)	1,117 (33.68)	
30–39 year-old	2,951 (25.63)	904 (27.25)	
40–49 year -old	2,569 (22.31)	653 (19.69)	
50–59 year -old	1,740 (15.11)	390 (11.76)	
Same or above 60 year-old	1,089 (9.46)	172 (5.19)	
Income quartile			0.19
Medical aid	2,300 (9.64)	672 (10.92)	
Poorest	1,093 (20.28)	357 (20.55)	
Poor	2,762 (24.36)	795 (24.31)	
Moderate	2,586 (22.8)	738 (22.57)	
Richest	2,599 (22.92)	708 (21.65)	
Year of HIV diagnosis			0.09
2004-2009	2,668 (23.17)	818 (24.66)	
2010-2014	3,469 (30.12)	1,014 (30.57)	
2015-2019	5,379 (46.71)	1,485 (44.77)	
City/District*			<0.001
Seoul	3,586 (31.34)	1,239 (37.48)	
Busan	887 (7.75)	230 (6.96)	
Daegu	450 (3.93)	113 (3.42)	
Incheon	670 (5.85)	239 (7.23)	
Gwangju	288 (2.52)	67 (2.03)	
Daejeon	281 (2.46)	80 (2.42)	
Ulsan	167 (1.46)	23 (0.7)	
Sejong	19 (0.17)	0 (0)	
Gyeonggi-do	2,594 (22.67)	688 (20.81)	
Gangwon-do	223 (1.95)	41 (1.24)	
Chungcheongbuk-do	305 (2.67)	60 (1.81)	
Chungchoengnam-do	389 (3.4)	89 (2.69)	
Jeollabuk-do	296 (2.59)	72 (2.18)	
Jeollanam-do	309 (2.7)	90 (2.72)	
Gyeongsangbuk-do	337 (2.94)	109 (3.3)	
GKyeongsangnam-do	513 (4.48)	129 (3.9)	
Jeju	130 (1.13)	37 (1.12)	

Data is presented as number (%)

*74 individuals had no residential data.

In 2014 and 2019, the incidence of syphilis was 20.2 and 15.1 per 1000 person-years, respectively (S2 Fig). This value increased slightly without statistical significance.

### Epidemiology of syphilis in the total population from the KDCA dataset

The KCDA dataset classified syphilis as a compulsory notifiable infectious disease, and 9,974 individuals were recorded in the syphilis surveillance data. After excluding 149 cases of congenital syphilis, 24 cases of leprosy, and seven cases diagnosed before 2014, the final study population consisted of 9,794 individuals (69.83% had primary syphilis (N = 6,839), and 30.17% had secondary syphilis (N = 2,955) (S3 Fig).

**Fig 1:**
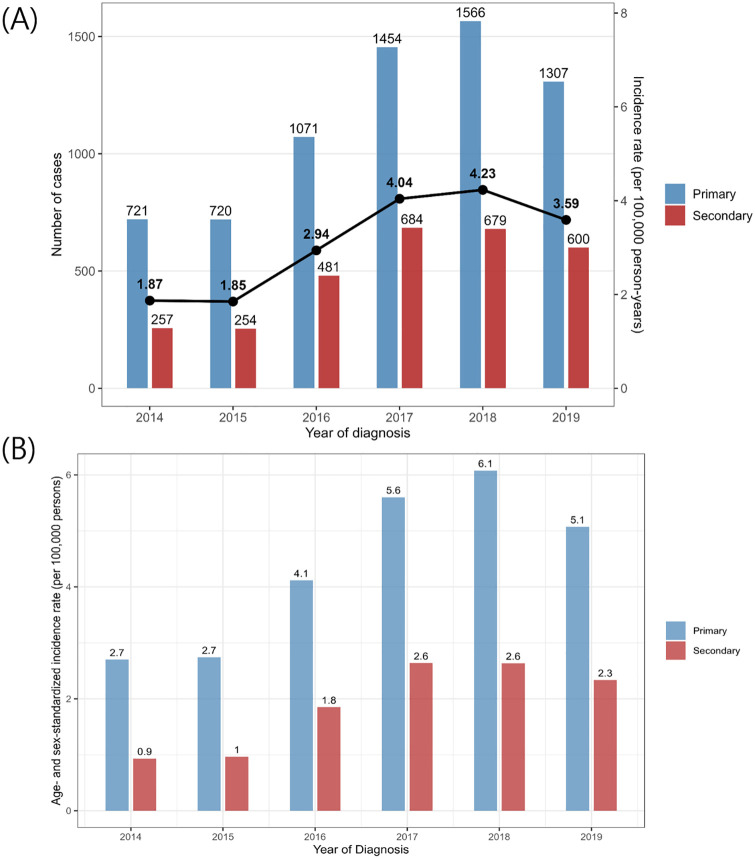
(A) The trend of syphilis cases in the total population (B) The trend of Age- and sex-standardized incidence rate (per 100,000 persons). The blue column indicates primary syphilis, while the red column indicates secondary syphilis. Black dot incidcates the incidence of overall syphilis per 100,000 population.

From 2014 to 2019, the number of reported primary and secondary syphilis cases showed distinct trends. Primary syphilis cases remained relatively stable from 2014 to 2015 (721 and 720 cases, respectively) but peaked in 2018 (1,566 cases) before declining to 1307 cases in 2019. In contrast, secondary syphilis cases exhibited a gradual increase, rising from 257 cases in 2014 to a peak of 684 cases in 2017, before slightly decreasing to 600 cases in 2019. The incidence of overall syphilis per 100,000 population followed a similar trend, increasing from 1.87 in 2014 to 4.23 in 2018, followed by a decline to 3.59 in 2019. The age- and sex-standardized incidence rate of primary syphilis reached its peak in 2018 (6.1 per 100,000 population), while the rate of secondary syphilis remained stable in 2017 and 2018 (2.6 per 100,000 population) (Fig 1).

### Comparison of PLWH and the total population from two datasets

Table 2 shows the demographic characteristics of patients with early syphilis in the PLWH cohort and the total population. Compared to the HIV group, the total population exhibited a higher proportion of males < 20 years old and those≥ 60 years old. Regionally, the proportion of syphilis cases among individuals with HIV was higher among males and females in Seoul, but lower in Gyeonggi-do, and Jeju.

**Table 2:** Epidemiology of people living with HIV and total population who were diagnosed with early syphilis.

	HIV		Total population	
	Male (N = 3,286)	Female (N = 31)	Male (N = 6,724)	Female (N = 3,076)
Age				
Below 20-year-old	79 (2.4)	2 (6.45)	514 (7.6)	464 (15.1)
20–29 year-old	1,107 (33.69)	10 (32.26)	2521 (37.5)	959 (31.2)
30–39 year-old	900 (27.39)	4 (12.9)	1574 (23.4)	563 (18.3)
40–49 year-old	650 (19.78)	3 (9.68)	1037 (15.4)	348 (11.3)
50–59 year-old	385 (11.72)	5 (16.13)	619 (9.2)	378 (12.3)
Same or above 60 year-old	165 (5.02)	7 (22.58)	459 (6.8)	364 (11.8)
City/District				
Seoul	1,233 (37.5)	6 (19.3)	1608 (23.9)	436 (14.2)
Busan	229 (7.0)	1 (3.2)	442 (6.6)	250 (8.1)
Daegu	112 (3.4)	1 (3.2)	255 (3.8)	189 (6.1)
Incheon	233 (7.1)	6 (19.3)	583 (8.7)	257 (8.4)
Gwangju	64 (1.9)	3 (9.7)	196 (2.9)	122 (4.0)
Daejeon	80 (2.4)	0 (0)	190 (2.8)	109 (3.5)
Ulsan	23 (0.7)	0 (0)	77 (1.1)	46 (1.5)
Sejong	1 (0.03)	0 (0.00)	25 (0.4)	13 (0.4)
Gyeonggi-do	682 (20.8)	6 (19.3)	1868 (27.8)	823 (26.8)
Gangwon-do	40 (1.2)	1 (3.2)	164 (2.4)	80 (2.6)
Chungcheongbuk-do	60 (1.8)	0 (0)	134 (2.0)	89 (2.9)
Chungchoengnam-do	89 (2.7)	0 (0)	192 (2.9)	125 (4.1)
Jeollabuk-do	72 (2.2)	0 (0)	130 (1.9)	92 (3.0)
Jeollanam-do	89 (2.7)	1 (3.2)	156 (2.3)	89 (2.9)
Gyeongsangbuk-do	104 (3.2)	5 (16.1)	261 (3.9)	138 (4.5)
Gyeongsangnam-do	128 (3.9)	1 (3.2)	281 (4.2)	160 (5.2)
Jeju-do	37 (1.1)	0 (0)	162 (2.4)	58 (1.9)

Data is presented as number (%)

The incidence rate ratios (IRRs) of the three variables were calculated using Poisson regression analysis (Table 3). HIV infection was associated with a 5.04-fold higher incidence rate of syphilis (IRR, 5.04; 95% Confidence interval (CI), 4.77–5.34; p < 0.0001). The IRR for the year was 1.15, indicating that the incidence of syphilis increased by 14.5% as the years elapsed (IRR, 1.15; 95% CI, 1.13–1.16; p < 0.0001). Males had a 2.4 times higher incidence rate of syphilis than females after adjusting for other factors in the model (IRR, 2.36; 95% CI, 2.26–2.46; p < 0.0001).

**Table 3:** Incidence rate ratios of early syphilis using Poisson regression.

Variables	Simple regression	Multiple Regression	p-value
HIV	6.12 [5.79-6.47]	5.04 [4.77-5.34]	<0.0001
Year	1.15 [1.13-1.16]	1.15 [1.13-1.16]	<0.0001
Male	2.56 [2.46-2.67]	2.36 [2.26-2.46]	<0.0001

Data is presented as number [95% Confidence Interval]

**Fig 2:**
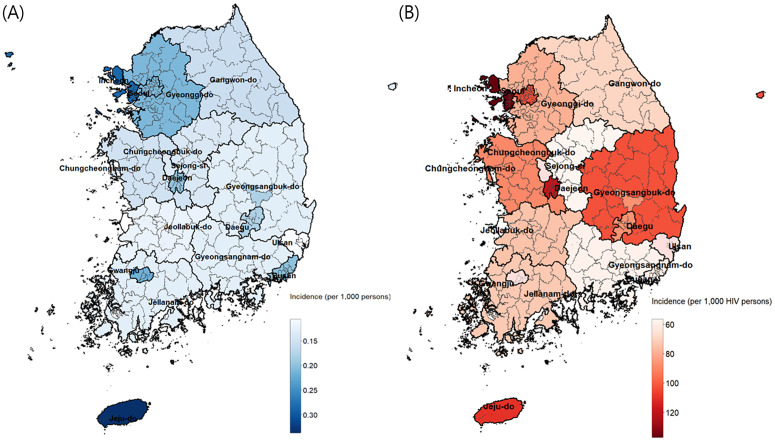
Geographical distribution of early syphilis (A) in the total population and (B) in people living with HIV.

In a map of the geographical incidence of syphilis by cities and districts, the incidence was highest in the Jeju, followed by Incheon, Gwangju, and Gyeonggi-do in the national population. In contrast, the incidence of syphilis among PLWH was highest in Incheon, followed by Daejeon, Jeju and Seoul (Fig 2).

**Fig 3:**
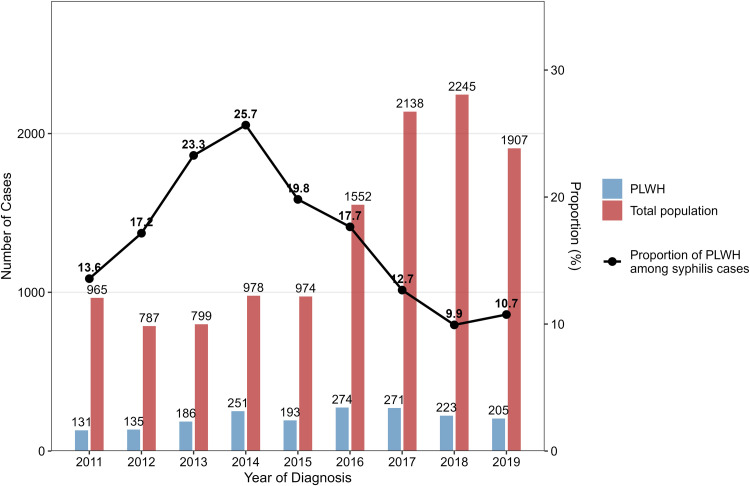
Comparison of time trend of early syphilis between PLWH and total population. Abbreviations: PLWH, people living with HIV.

Fig 3 shows the number of reported syphilis cases in the total population and the PLWH cohort, as well as the proportion of syphilis cases occurring in PLWH. The proportion of PLWH among individuals with early syphilis increased from 13.6% in 2011, peaked at 25.7% in 2014, and subsequently declined to 10.7% in 2019. Notably, after 2015, a sharp increase was observed in the number of syphilis cases in the total population, whereas the number of syphilis cases among PLWH remained stable.

Choropleth maps at the provincial level of the incidence rates per 1,000 population for each region. Color gradients were applied to represent varying incidence levels, highlighting geographical differences in syphilis rates.

## Discussion

In this study, we examined the epidemiology of syphilis in the Republic of Korea and compared its epidemiological characteristics between PLWH and those without HIV using two separate datasets. HIV and syphilis are closely linked; however, advancements in antiretroviral therapy have significantly mitigated syphilis susceptibility associated with immune suppression. In our study, an increase in HIV prevalence was associated with a 5.04-fold increase in the incidence of syphilis, indicating a strong positive association between presence of HIV and early syphilis.

Previous studies among PLWH have identified younger age, MSM status, multiple sexual partners, and unprotected sex as significant predictors of incident syphilis [20–22]. Notably, some PLWH with high HIV viral loads or longer duration since HIV diagnosis had higher rates of syphilis diagnosis [23]. However, another study revealed that a shorter time since HIV diagnosis was associated with incident syphilis [24]. In our study, PLWH who were diagnosed with HIV at a younger age were more likely to have a higher incidence of early syphilis. Although a longer duration since HIV diagnosis was associated with a higher incidence of early syphilis, the association was not statistically significant.

MSM have a high burden of syphilis with the global pooled prevalence of 7.5% [25]. In Germany, a 19% increase in syphilis cases in 2015 was attributed to a rise in reported cases among MSM across all age groups [26]. Due to social discrimination and homophobia, MSM may be less likely to disclose their sexual orientation, potentially leading to suboptimal syphilis screening in this population [27]. In our study, we were unable to extract data on sexual orientation from the National Health Insurance dataset. However, considering that MSM account for over 70% of PLWH in Korea [28], and that the proportion of men with a history of syphilis among individuals who are HIV-positive is higher than that among the overall population with syphilis, it can be inferred that the epidemiology of syphilis through MSM sexual networks is likely similar. As in multiple studies from other countries, MSM in Korea are expected to disproportionately bear the burden of syphilis.

In our study, the proportion of PLWH among the early syphilis cases in the total population declined. Furthermore, the geographic distributions of syphilis cases differed between the national population and PLWH. In Japan, PLWH accounted for 5.9% of newly diagnosed syphilis cases in 2016 [29], which is substantially lower than that the proportion observed in the Republic of Korea (20.85%). However, the number of syphilis cases in Japan increased by 36.0% by 2021 [30]. Despite this increase, the proportion of PLWH among early syphilis cases has shown a declining trend [29]. In addition, there has been an increase in syphilis cases among older adults aged ≥ 60 years [29,31]. Therefore, HIV status alone does not fully explain the current surge. This infection may have become prevalent outside the sexual networks of individuals with HIV or MSM.

From this perspective, the decision to revert to a universal surveillance system in 2024 is commendable as many other countries continue to maintain universal reporting of syphilis [32,33]. With this system, all detected cases will be reported, providing a comprehensive and objective overview of the country’s syphilis epidemic and facilitating the development of targeted prevention and management strategies.

Currently, there is no vaccine or pre-exposure prophylactic treatment for syphilis. Hence, early diagnosis and prompt treatment are essential. Since the early phase of syphilis can be asymptomatic, it is essential to educate individuals at high risk of syphilis and broaden their access to testing and clinics. Also, screening for syphilis in adolescents and adults who have been sexually active is highly recommended [34].

A limitation of this study is that despite syphilis being designated as a notifiable infectious disease under mandatory surveillance, syphilis cases among PLWH were identified based on medication and diagnostic codes. Therefore, not all cases of early syphilis may have been included in the national population, especially early latent syphilis, potentially resulting in an underestimation of syphilis cases. Infectious diseases specialists who usually care for PLWH often check syphilis tests precisely and symptoms closely with the prescription of antiretroviral therapy. This differential testing intensity may lead to the IRR gap. In addition, we restricted our analyses to cases classified as primary or secondary syphilis to ensure comparability between the two datasets. Therefore, congenital and late syphilis cases were not included in this study, and our findings do not represent the overall epidemiology of syphilis. Nevertheless, analyzing these two datasets together and conducting geographical analyses can provide valuable insights into future strategies for syphilis prevention and control. Lastly, data on sexual orientation or sexual behavior were not collected; therefore, MSM-specific inferences could not be drawn from our datasets. Given that MSM constitute a key population for HIV infection in Korea, trends in syphilis among PLWH may indirectly reflect the vulnerability of MSM to new syphilis infections. Future epidemiologic studies should include information on sexual orientation and behavioral characteristics to better understand the dynamics of syphilis transmission.

In conclusion, although syphilis is prevalent among PLWH, the epidemiology of the total population and PLWH revealed different patterns, highlighting the need for multifaceted policies to prevent syphilis transmission.

## Supporting information

S1 Fig 1. Flow of study population from National Health Insurance Service. (DOCX)

S2 Fig 2. Incidence of syphilis in people living with HIV. The incidence of syphilis was calculated as the number of syphilis cases per 1000 person-years among people living with HIV in the NHIS dataset.
(DOCX)

S3 Fig 3. Flow of study population from the KDCA dataset. (DOCX)
