# Supplementary material for: Characteristics and geographical distribution of syphilis among people with human immunodeficiency virus and the National Population in Republic of Korea
Source: PLoS One. 2026 Mar 26;21(3):e0340324. doi: 10.1371/journal.pone.0340324 (PMC13020971; doi:10.1371/journal.pone.0340324)
Supplement: S1 Fig — (DOCX) [file pone.0340324.s001.docx]

**Supplementary Figure 1. Flow of study population from National Health Insurance Service**

|  | | **Eligible individuals**  N=20,423 | | | |  | | |  | | | |
| --- | --- | --- | --- | --- | --- | --- | --- | --- | --- | --- | --- | --- |
|  | |  | |  | |  | | |  | | | |
|  | |  |  |  | | **Excluded from analysis:**  Patients for whom the V103 diagnostic code appears only once : 2,350  2002-2004 washout period: 1,073 | | | | | | |
|  | |  |  |  | |  |  |  |  |  |  |  |
|  | |  |  |  | |  | | |  | | | |
|  | | **Defined individuals**  N=17,000 | | | |  | | |  | | | |
|  | |  | |  | |  | | |  | | | |
|  | |  |  |  | | **Excluded from analysis:**  The first diagnosis year of HIV is not between 2004 and 2019: 1,861 | | | | | | |
|  |  |  |  |  | |  |  |  |  |  |  |  |
|  | |  |  |  | |  | | |  | | | |
|  | **Final individuals**  N=14,833 | | | | | |  | |  | | | |
|  | |  |  |  | |  | | |  | | | |
|  | |  |  |  | |  | | |  | | | |
| **PLWH without syphilis^*^**  11,516 (77.64%) | | | | |  | | **PLWH with syphilis**^†^  3,317 (22.36%) | | | | | |
|  | | | | |  | |  |  |  |  |  |  |
|  | | | | |  | | Before HIV diagnosis  1,373 (41.39%) | |  |  | After HIV  diagnosis  1,944 (58.61%) | |
|  | | | | |  | |  | | | | | |

Abbreviations: PLH, people living with HIV

^*^Footnote: The group of PLWH without syphilis includes 306 individuals who were not classified as having syphilis according to the study definition.

^†^Syphilis was defined as having an ICD-10 code of A50, A51, or A53, accompanied by a concurrent prescription of either benzathine penicillin G (single dose; *n* = 3,218, 97.0%), doxycycline (14-day course; *n* = 92, 2.8%), or ceftriaxone (1 g for 10–14 days; *n* = 7, 0.2%).
