# Supplementary material for: Characteristics and geographical distribution of syphilis among people with human immunodeficiency virus and the National Population in Republic of Korea
Source: PLoS One. 2026 Mar 26;21(3):e0340324. doi: 10.1371/journal.pone.0340324 (PMC13020971; doi:10.1371/journal.pone.0340324)

**Supplementary Figure 2. Incidence of syphilis in people living with HIV**

The incidence of syphilis was calculated as the number of syphilis cases per 1000 person-years among people living with HIV in the NHIS dataset.


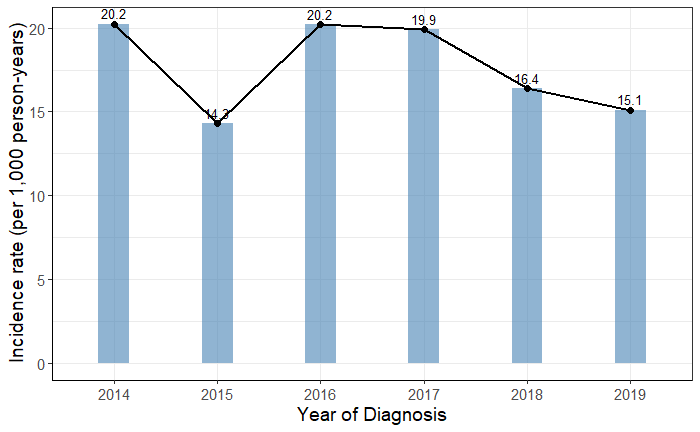

Supplement: S2 Fig — The incidence of syphilis was calculated as the number of syphilis cases per 1000 person-years among people living with HIV in the NHIS dataset. (DOCX) [file pone.0340324.s002.docx]
