# Supplementary material for: Characteristics and geographical distribution of syphilis among people with human immunodeficiency virus and the National Population in Republic of Korea
Source: PLoS One. 2026 Mar 26;21(3):e0340324. doi: 10.1371/journal.pone.0340324 (PMC13020971; doi:10.1371/journal.pone.0340324)
Supplement: S3 Fig — (DOCX) [file pone.0340324.s003.docx]

**Supplementary Figure 3. Flow of study population from the KCDA dataset**

|  | | **Eligible individuals**  N=9,974 | | | | |  |  | |  |
| --- | --- | --- | --- | --- | --- | --- | --- | --- | --- | --- |
|  | |  | | |  | |  | **Excluded from analysis:** 180  Congenital syphilis: 149  Leprosy: 24  Diagnosed before 2014: 7 | | |
|  | |  | |  |  | |  |  |  |  |
|  | |  | |  |  | |  |  | |  |
|  | | **Final individuals (2014-2019)**  N=9,794 | | | | |  |  | |  |
|  | |  | |  |  | |  |  | |  |
|  | |  | |  |  | |  |  | |  |
|  | **Primary syphilis**  N=6,839 (69.83%) | |  |  |  | **Secondary syphilis**  N=2,955 (30.17%) | | |  |  |
